# Versatilely tuned vertical silicon nanowire arrays by cryogenic reactive ion etching as a lithium-ion battery anode

**DOI:** 10.1038/s41598-021-99173-4

**Published:** 2021-10-05

**Authors:** Andam Deatama Refino, Nursidik Yulianto, Iqbal Syamsu, Andika Pandu Nugroho, Naufal Hanif Hawari, Alina Syring, Evvy Kartini, Ferry Iskandar, Tobias Voss, Afriyanti Sumboja, Erwin Peiner, Hutomo Suryo Wasisto

**Affiliations:** 1 Institute of Semiconductor Technology (IHT), Technische Universität Braunschweig, Hans-Sommer-Straße 66, 38106 Braunschweig, Germany; 2 Laboratory for Emerging Nanometrology (LENA), Technische Universität Braunschweig, Langer Kamp 6, 38106 Braunschweig, Germany; 3 Engineering Physics Program, Institut Teknologi Sumatera (ITERA), Jl. Terusan Ryacudu, Way Huwi, Lampung Selatan, Lampung, 35365 Indonesia; 4 Research Center for Physics, National Research and Innovation Agency (BRIN), Jl. Kawasan Puspiptek No. 441-442, South Tangerang, 15314 Indonesia; 5 Research Center for Electronics and Telecommunication, National Research and Innovation Agency (BRIN), Jl. Sangkuriang-Komplek LIPI Gedung 20, Bandung, 40135 Indonesia; 6 Material Science and Engineering Research Group, Faculty of Mechanical and Aerospace Engineering, Institut Teknologi Bandung, Jl. Ganesha 10, Bandung, 40132 Indonesia; 7 Center for Science and Technology of Advanced Materials, National Nuclear Energy Agency (BATAN), South Tangerang, 15314 Indonesia; 8 Department of Physics, Faculty of Mathematics and Natural Science, Institut Teknologi Bandung, Jl. Ganesha 10, Bandung, 40132 Indonesia; 9 PT Nanosense Instrument Indonesia, Umbulharjo, Yogyakarta 55167 Indonesia

**Keywords:** Nanowires, Batteries

## Abstract

Production of high-aspect-ratio silicon (Si) nanowire-based anode for lithium ion batteries is challenging particularly in terms of controlling wire property and geometry to improve the battery performance. This report demonstrates tunable optimization of inductively coupled plasma reactive ion etching (ICP-RIE) at cryogenic temperature to fabricate vertically-aligned silicon nanowire array anodes with high verticality, controllable morphology, and good homogeneity. Three different materials [i.e., photoresist, chromium (Cr), and silicon dioxide (SiO_2_)] were employed as masks during the subsequent photolithography and cryogenic ICP-RIE processes to investigate their effects on the resulting nanowire structures. Silicon nanowire arrays with a high aspect ratio of up to 22 can be achieved by tuning several etching parameters [i.e., temperature, oxygen/sulfur hexafluoride (O_2_/SF_6_) gas mixture ratio, chamber pressure, plasma density, and ion energy]. Higher compressive stress was revealed for longer Si wires by means of Raman spectroscopy. Moreover, an anisotropy of lattice stress was found at the top and sidewall of Si nanowire, indicating compressive and tensile stresses, respectively. From electrochemical characterization, half-cell battery integrating ICP-RIE-based silicon nanowire anode exhibits a capacity of 0.25 mAh cm^−2^ with 16.67% capacity fading until 20 cycles, which has to be improved for application in future energy storage devices.

## Introduction

Nowadays, the emerging trends in electric vehicles and global shift to the use of renewable energy require high-performing energy storage devices (i.e., batteries) with high capacity and long cycle life. Due to its high theoretical capacity, silicon (Si) anode has been intensively investigated to be a game-changer in battery applications overcoming the limitations of the most commercially used carbon-based anodes^1,2^. Particularly in lithium-ion batteries, alloying of lithium into Si enables energy storage of almost ten times compared to the intercalation of lithium into graphite^3^. However, by its nature, silicon possesses several drawbacks, in which it undergoes extreme volume change of up to 300% upon charging/discharging, becomes pulverized, and causes an extreme degradation of capacity in a low number of cycles^4^. Si nanostructures are proposed to overcome this cycling challenge whilst at the same time possess a potential to increase the rate performance due to the increase of surface to volume ratio^1,5^. Amongst the various geometries of Si nanostructures, nanowire has been favorable because it can withstand the volume change upon cycle as well as facilitate efficient one-dimensional transport of charge carriers^6,7^. Several studies have reported Si-nanowire-based anodes for lithium-ion batteries with excellent performance^3,8,9^.

Vertical Si nanowires can be fabricated by either bottom-up or top-down techniques. The bottom-up fabrication techniques include the growth of Si nanowires via vapor–liquid-solid (VLS) and supercritical fluid-liquid–solid (SFLS) mechanisms^10–12^. Despite the fact that long nanowires of up to 10 µm with smooth sidewalls could be produced, this method still suffered from low controllability of the nanowire homogeneity and alignment. On the other hand, the top-down fabrication approaches offer more controllable results. Among the available chemical-based wet processes, metal-assisted chemical etching (MACE) has been often used to manufacture high-aspect-ratio Si-nanowire-based anodes for lithium-ion batteries^13–15^. Here, a pre-patterned metal thin film is normally deposited on top of the wafer to determine the wire diameter, whilst the processing time is controlled to define the wire length.

High conductivity of the anode is desirable for battery performance. It was demonstrated that heavily-*n*-doped Si nanowires could exhibit not only high conductivity, but also ultrafast charging compared to their intrinsic counterparts^16^. However, when they were fabricated using the MACE method, a drawback in terms of balancing the possible amount of dopants in Si nanowires and the resulting porosity was revealed^13^. Since the doping atoms act as nucleation sites for pore formation on the Si nanowire surface during chemical etching, higher doping concentrations consequently yield more porous structures. This porosity is beneficial to increase the active surface area and provide buffer space during the volume change of Si. Nevertheless, excessive porosity in heavily doped Si nanowires facilitates more oxide formation, leading to lower battery performance.

To overcome excessive pore formation in MACE, a top-down dry etching technique using ICP-RIE is proposed to produce vertically aligned Si nanowire arrays with smooth sidewalls and a high aspect ratio independent of doping concentration^17–19^. Here, instead of using wet chemical solutions, a plasma containing ions and radicals is employed as an etchant to pattern the Si wafer. Compared to widely used top-down method such as wet chemical based etching, this method has the advantage of its unnecessity to use hazardous substances such as high concentration HF to etch the silicon. Moreover, although the wet chemical based etching is simpler and requires relatively short processing steps, it is still not used in a large scale fabrication of Si nanowire anodes until today, which is also a concern in future battery productions. In contrast, although this method is rather complicated, it has been widely used in many large-scale industrial semiconductor processing lines^20,21^, showing its potential to be integrated to a large-scale battery production.

Bosch and cryogenic etching processes are established as the most commonly used ICP-RIE techniques to realize Si nanowire arrays. The Bosch approach works by consecutively altering surface passivation and isotropic etching to achieve profiles of high aspect ratio. Although high-aspect-ratio nanowires with good verticality can be obtained, this method adversely provides a phenomenon so-called scalloping effect, where the nanowires possess choppy sidewalls due to the repeatedly alternating passivation and etching steps^22^. Moreover, the passivation steps of Bosch process also have the consequence that a polymer layer is built on the sidewalls, which needs to be removed after the etching process^23^. Meanwhile, passivation and etching processes can take place simultaneously during ICP-RIE at cryogenic temperatures yielding smooth sidewalls without the need to remove passivation layer after etching. This technique is made possible by the low (cryogenic) process temperatures, at which a passivation layer of SiO_x_F_y_ is formed to hamper chemical etching of Si^24^. Only the areas perpendicular to ion bombardment undergo physical etching, where F radicals can ruin the SiO_x_F_y_ layer and subsequently remove the Si atoms^25^. Because of these simultaneous reactions, smooth sidewall and homogeneous geometry of the nanowires can be achieved by controlling the process parameters. After the process, the passivation layer becomes volatile and desorbs from the Si surface along with the warm-up from cryogenic to room temperature, eliminating the need for passivation layer removal^26^.

Regardless of several reports on the fabrication of vertical high-aspect-ratio Si nanowires using cryogenic ICP-RIE combined with several nanolithography methods (i.e., optical ultraviolet (UV) lithography^17,27,28^, nanoimprint lithography (NIL)^29,30^, electron beam lithography (EBL)^31^, and colloidal nanosphere lithography^32,33^), the resulting nanowires had not been employed as lithium-ion battery anodes and their mechanical characteristics were not investigated to identify the stress inside the structures (i.e., either compressive or tensile stress). Moreover, the effect of mask materials on the produced nanowires using the same wafers was still unknown.

In this work, we report on tunable top-down fabrication and optimization of high-aspect-ratio vertical Si nanowire anodes for lithium-ion batteries by combining cryogenic ICP-RIE and photolithography. During the patterning process, three different mask materials [i.e., photoresist, chromium, and silicon dioxide (SiO_2_)] were attempted on the identical Si wafers to investigate their effects on the etching behaviors. The etching recipes were optimized by adjusting several key parameters (e.g., temperature, pressure, plasma content, ICP power, and high-frequency power), in which the defects in nanowires and the strategy to achieve high-aspect-ratio structures were discussed. Besides, Raman spectroscopy was employed to gain insights into the mechanical property of the Si nanowires (i.e., their stress condition) before and after detachment from the substrate. Fabricated Si nanowire anodes were integrated into half-cell lithium-ion batteries to evaluate their electrochemical performance. We treated the electrochemical testing presented in this article as a preliminary result, in which Si nanowires with relatively low aspect ratio were incorporated. Although the morphology of the tested Si anode was not optimized, its typical cycling behavior could be observed and analyzed with respect to the corresponding Si nanowire geometry. Further strategies to improve anode performance are also suggested.

## Results and discussion

### Processing routes for different mask materials

**Figure 1:**
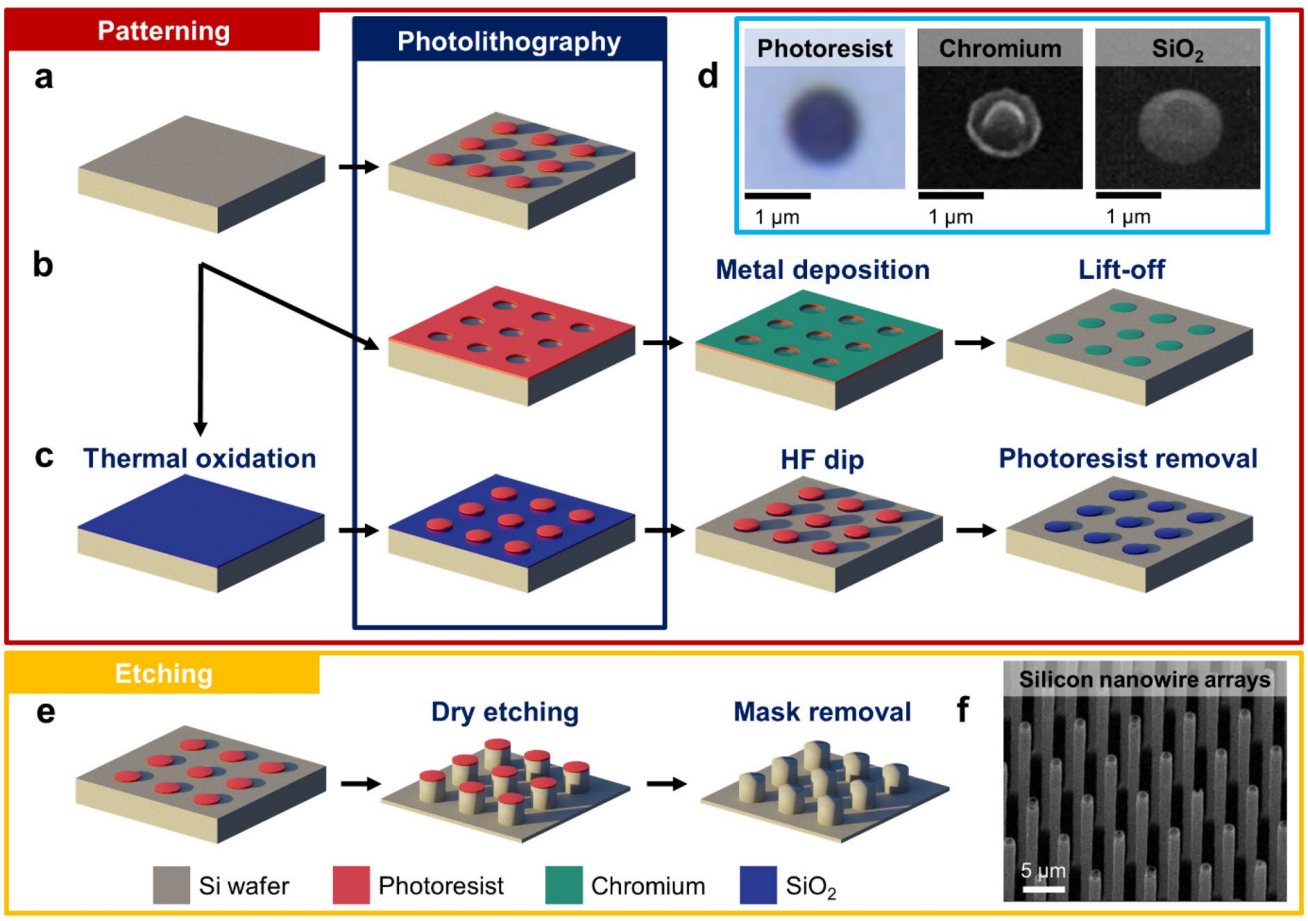
Fabrication routes of vertical silicon (Si) nanowire arrays using different mask materials. Patterning of (**a**) photoresist, (**b**) chromium (Cr), and (**c**) silicon dioxide (SiO_2_) masks combines photolithography and several thin-film fabrication techniques (i.e., electron-beam evaporation for Cr thin film and wet thermal oxidation for SiO_2_ thin film). (**d**) The resulting photoresist, Cr, and SiO masks on Si substrates showing circular patterns with a diameter of ~1 µm. (**e**) Inductively coupled plasma reactive ion etching (ICP-RIE) process of pre-patterned Si substrate yielding vertically aligned Si nanowire arrays. (**f**) Scanning electron micrograph of the fabricated vertical Si nanowire arrays.

Top-down fabrication of Si nanowire arrays begins with mask patterning. Here, we used photolithography considering its flexibility to modify the integrated thin-film-based masks. Figure 1a–c illustrate the processing steps involving photolithography, thin-layer deposition, and etching to realize the patterned masks made of photoresist, Cr, and SiO_2_, respectively. The resulting circular masks made of those three materials, each with a diameter of ~1 µm, are depicted in Fig. 1d. For the first type of mask (i.e., photoresist), diluted AZ 5214 E resist in AZ EBR (1:1) was employed. It was first spin-coated on top of a Si wafer and subsequently soft-baked to remove the remaining solvent. Then, one-step exposure was conducted to transfer circular patterns from a light-field mask to the photoresist. The exposed area could be removed using developer solution, leaving arrays of circular photoresist (see Fig. 1a,d, left). It should be noted that the diameter of resulting resist pattern was always less than that of the used mask template due to the diffracted light during exposure.

For the second mask template, we employed a 100 nm thick Cr layer as a hard mask because of its high selectivity in Si deep reactive ion etching (DRIE)^34^. This material was also proven to be able to sustain as a mask during deep physical RIE of ~5 µm high vertical gallium nitride (GaN) nanowires, in which GaN is known as a wide-bandgap semiconductor possessing higher hardness, stiffness (i.e., Young’s modulus of 300–350 GPa), and mechanical stability than Si^33,35–38^. Besides, considering the required later fabrication process (e.g., transfer of Si nanowires to another metal substrate), when the top electrical contact has to be created in the final device, the deposition of a Cr thin film during the initial patterning of Si nanowires is beneficial to simplify the metallization step. Hence, additional polymer filling or planarization prior to metal deposition like in common nanodevices having vertical architecture is not needed^39–41^. Furthermore, due to its good adhesion to Si and SiO_2_, Cr has been frequently used as an adhesion layer for less-adhesive metals [e.g., gold (Au)]^42–45^. Figure 1b shows the Cr mask formation sequence involving photolithography, electron-beam evaporation, and metal lift-off. During the photolithography process, the image has to be inverted. Here, an additional image-reversal baking step was added right after the first exposure, followed by a flood exposure of UV light onto the whole wafer area. This extra step changed the resist chemistry and made the initially exposed area become less soluble. Once the sample had been dipped in the developer solution, the freely exposed silicon surfaces having circular pattern could then be created. Finally, after electron-beam evaporation of a Cr thin film and its subsequent lift-off had been carried out, the patterned Cr mask arrays were realized on the Si substrate (see Fig. 1d, middle).

Besides photoresist and Cr masks, we fabricated another hard pattern made of ~130 nm thick SiO_2_ as the third mask variant. To grow a SiO_2_ layer on a Si wafer, thermal oxidation at 1100 °C was employed because of its simplicity and feasibility to produce a cleaner interface between those two materials (i.e., SiO and Si) than typical chemical vapor deposition (CVD) at a lower temperature (e.g., 550 °C)^46–48^. Next, a standard one-step photolithography process was conducted to form a photoresist pattern on top of the SiO_2_ surface (see Fig. 1c). To transfer the pattern down to the SiO_2_, the sample was dipped into hydrofluoric acid (HF) solution, in which the HF reacts with the freely exposed oxide and etch it isotropically. Due to the large diameter of the photoresist pattern compared to the thickness of the SiO_2_ layer (~10:1), an optimum dipping duration was found to be 2 min to strip the uncovered SiO_2_ layer completely without removing photoresist layer on the wafer. This process was then followed by photoresist removal using acetone. As a result, circular SiO_2_ masks with a positive sidewall slope and a flat top surface could be realized on Si substrate (see Fig. 1d, right). This cone shape not only indicates that HF reacts more at the interface between the SiO_2_ and the photoresist during the wet etching, but also affects the later Si dry etching behavior.

Following the mask creation, cryogenic dry etching was performed to realize vertically aligned Si nanowire arrays as illustrated in Fig. 1e. The pre-patterned sample was put into an ICP-RIE chamber that was cooled down to cryogenic temperature. Using a combination of O_2_ and SF_6_ gases, plasma was generated inside the chamber. The gases were thereby dissociated into radicals and ions and transported into the sheath at the vicinity of the sample surface where the etching occurred. Due to the presence of patterned mask in some areas of the sample, etching behaves differently on the surface. The three employed mask materials result in different etch rates. Nevertheless, the etch rate of Si in each of these cases is much higher compared to that of the mask material. Hence, the Si could be etched selectively. After the first etch of Si surface, a passivation layer was formed on the sidewall surface below the mask, protecting the underlying Si from etching. This phenomenon propagated with the progressing of the Si etching process, resulting in the formation of vertical Si nanowire arrays (see Fig. 1f). As soon as the temperature increased to room temperature, the passivation layer desorbed from the sidewalls. After the etching process had been terminated, the masks of photoresist, Cr, and SiO_2_ could be removed from the top of the nanowires using acetone, Cr etchant, and HF, respectively.

### Tuning on nanowire geometry by cryogenic reactive ion etching

To understand their effect on wire geometry and obtain good control of sidewall profile, several key ICP-RIE parameters (i.e., temperature, oxygen content, pressure, radio-frequency (RF) power, and ICP power) were optimized during anode fabrication. Here, photoresist masks with a diameter of 1 µm and a pitch of 4 µm were used as masks considering their processing simplicity, while etch time was set constant at 5 min for all samples to yield comparable results. The resulting nanowires were evaluated by their lengths and sidewall angles. Nanowire with perpendicular sidewall has sidewall angle of 90°, while those with cone and inverted cone shapes are represented with sidewall angles of > 90° and < 90°, respectively (see Figure S1 in the Supplementary Information).

**Figure 2:**
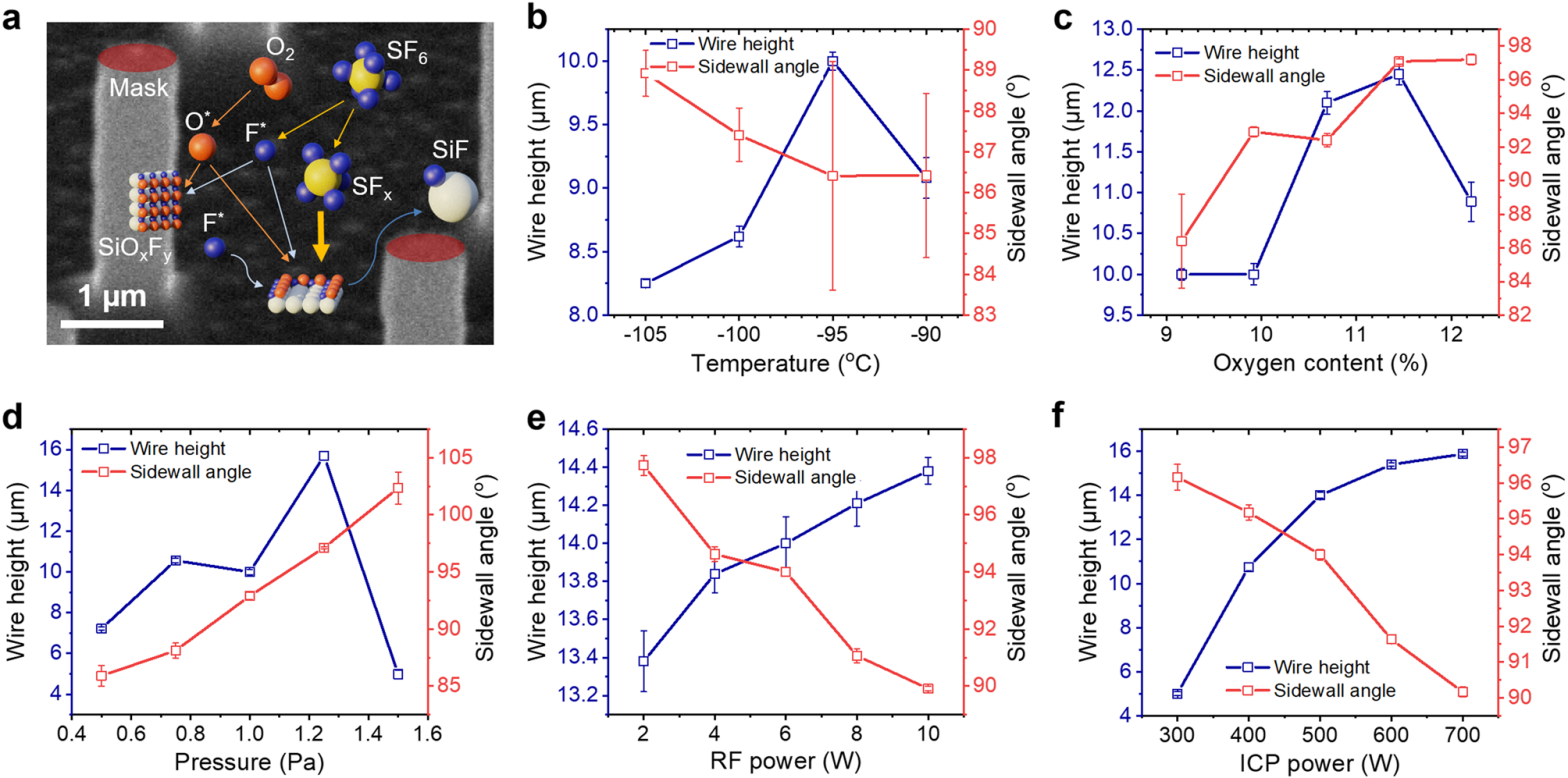
Etching mechanism and effects of the ICP-RIE processing parameters on the nanowire height and sidewall angle. (**a**) Scanning electron microscopy (SEM) image illustrating chemical species involved in the cryogenic dry etching process. Oxygen (O^*^) and fluorine (F^*^) form a passivation layer (SiO_x_F_y_) on the nanowire sidewall as well as at the bottom part of Si at cryogenic temperatures. The physical bombardment of SF_x_ ions triggered by the applied bias results in the removal of the bottom passivation layer. F radicals can react chemically with Si atoms at the bottom part forming volatile SiF_x_ species, which are detached from the crystal, resulting in anisotropic etching. In this case, the mask at the top of the nanowire (in red color) acts as a shielding layer to prevent both physical and chemical etching processes. Various parameters, namely (**b**) temperature, (**c**) oxygen content, (**d**) pressure, (**e**) radio-frequency (RF) power, and (**f**) ICP power affect the height and sidewall angle of Si nanowires.

The cryogenic dry etching mechanism on the surface of a patterned Si nanowire with a diameter down to 830 nm is explained in Fig. 2a. Within the generated ICP, O_2_ and SF_6_ gases were dissociated to O and F radicals (O^*^ and F^*^), SF_x_ ions, and electrons. The negatively charged radicals and ions were attracted to the surface by the positively-biased substrate. Both O^*^ and F^*^ were then adsorbed on the Si surface, forming an SiO_x_F_y_ layer that was stable at cryogenic temperatures. The vertically accelerated SF_x_ ions bombarded the surface with high kinetic energy and physically etched the passivation layers. Subsequently, F^*^ was chemically bonded to the exposed Si atom at the surface and detached as SiF_x_ (i.e., the etching process occurred). At the same time, the mask-covered Si areas were protected and did not undergo this etching process. Consequently, the removal of Si in the non-masked areas introduced sidewalls around the masked areas. While the passivation layer was continuously formed on all Si surfaces, the bombardment of ions mainly affected the areas perpendicular to the vertical ion bombardment direction. Thus, a passivation layer was built on the sidewall, resulting in a protection of Si against F^*^ etching. Depending on the processing duration, Si nanowires could be realized and adjusted according to the desired length. However, it should be noted that the possible length that could be achieved is limited by the aspect ratio dependent etching (ARDE) effect^49^. After the process had been completed, the sample temperature was gradually increased to room temperature and the SiO_x_F_y_ layers desorbed from the surface.

**Figure 3:**
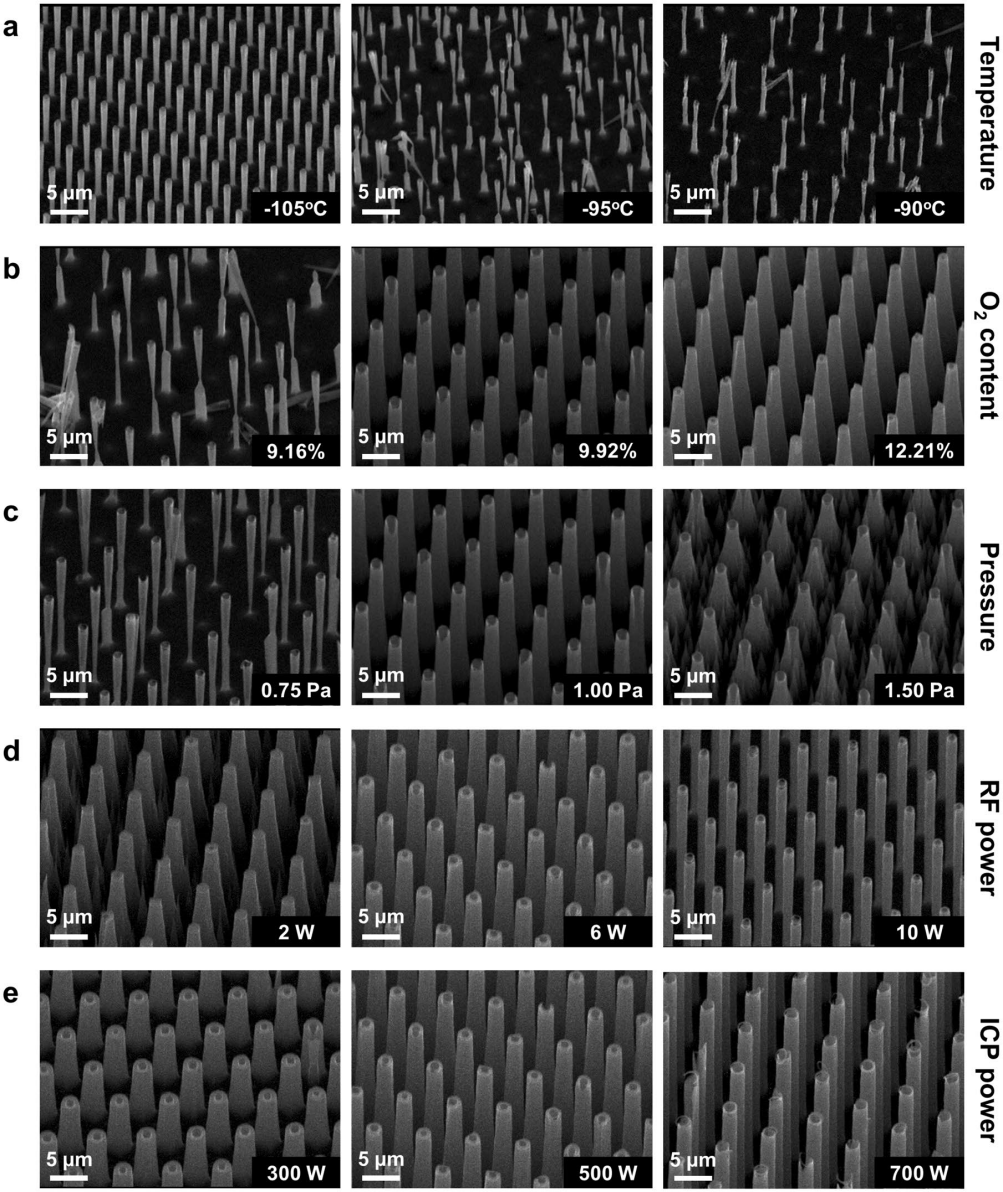
Vertical Si nanowire arrays fabricated by cryogenic ICP-RIE with different process parameters. The resulting Si nanowire profiles are affected by changing (**a**) temperature, (**b**) oxygen content, (**c**) pressure, (**d**) RF power, and (**e**) ICP power. Each of those parameters was varied while keeping the other parameters constant at the default parameter (see “Methods”). The increase in temperature, oxygen content, and pressure, respectively, led to more positively-inclined sidewalls. On the contrary, the rises of RF and ICP powers could remove the positive sidewalls yielding higher nanowires.

Temperature plays a vital role in cryogenic etching because it determines the formation and removal of the SiO_x_F_y_ passivation layer on the Si surface^26,50^. Figure 2b shows the dependence of nanowire height and sidewall angle on processing temperature. It is clear that lowering temperature to the point below −95 °C has a significant impact on increasing sidewall angle towards 90°. At the same time, it might also decrease the nanowire height, which corresponds to a lower etch rate. Both increasing sidewall angle and decreasing etch rate indicate stronger passivation. This can be attributed to the reaction kinetics around the sidewalls, where the balance between formation and removal of passivation layer is moved towards the former. Nonetheless, towards higher temperatures (−90 °C) the opposite trend was revealed, where a lower etch rate was again observed. Convergence of sidewall angle to the value of ~86.4° was found at temperatures of > −95 °C. It should be noted that more isotropic contribution to etching at higher temperatures can result in a collapse of nanowires, as the bottom part of the nanowire having a “beaver-bite” shape could not sustain from the ion bombardment (see Fig. 3a).

While temperature determines the stability of passivation layer, O_2_ content provides the amount of oxygen atoms that adsorb on the Si surface to form the SiO_x_F_y_ passivation layer. Here, it is represented by the percentage of O_2_ flow rate relative to the total gas flow rate containing O_2_ and SF_6_. The total gas flow rate itself was set to be constant. From Fig. 2c, rising O_2_ content from 9.16 to 12.21% revealed a highest etch rate of 2.42 µm/min at an O_2_ content of around 10.69%, where the nanowire height reached a length of 12.1 µm after being etched for 5 min. Simultaneously, the sidewall angle increased, while its value reached 90° when the O_2_ content was set between 9.16 and 9.92%. Thus, applying a higher O_2_ content (> 9.92%) will lead to both higher etch rate and sidewall angle of > 90° (see Fig. 3b). In this case, the bottom diameters of the developing nanowires will increase and etching will stop at the point when the surface of neighboring nanowires becomes in touch. Furthermore, in this regime, black Si will form on the bottom surface due to an over-passivation by oxygen^51,52^. Therefore, for high-aspect-ratio Si nanowires, maintaining a sidewall angle of 90° is more preferable than reaching the highest possible etch rate. Thus, in our further experiments, O_2_ content was maintained at ~9.92%.

During the cryogenic etching process, the chamber pressure is a parameter that affects the ionic angular distribution (IAD)^53^. Higher chamber pressure increases the collision rate between ions and thus reduces the directionality of ion bombardment. At low pressure, the ion can easily reach the bottom of Si surface. Hence, passivation layer removal at the bottom is more effective. However, some ions also have the probability to bounce back and etch the nanowire sidewalls resulting in a negative sidewall profile (i.e., a sidewall angle of < 90°) (see Fig. 2d). Meanwhile, higher pressure can increase the Si nanowire sidewall angle. This phenomenon is due to the less effective passivation layer removal, which is associated with the lower directionality of the ions or a broader IAD. Here, more collisions among the ions occur, leading to their difficulty to reach the Si bottom part. A balance between high directionality and minimum backscattering of ions could be achieved at a pressure of 1 Pa, which led to a sidewall angle of almost 90°. In terms of its effect on nanowire height, increasing pressure produces higher structures following a proportional trend. Nonetheless, similar to the condition when too high oxygen content was involved in the process, cone Si structures with a height of 4.98 µm were obtained at 1.5 Pa due to the reduced ion bombardment efficiency (see Fig. 3c, right).

Since physical etching is a determining factor in cryogenic Si etching, RF power, which leads to a self-bias of the sample holder, also influences the nanowire profile. This bias voltage attracts ions down to the sample surface and causes the bombardment that physically etches the passivation layer and the silicon surface. Furthermore, increasing RF power resulted in higher nanowires, i.e., etch rates (see Fig. 2e). This was due to the higher kinetic energy and momentum transferred by ions to the silicon surface. Higher RF power produced not only higher nanowire structures, but also lower sidewall angles because of the higher vertical momentum of the ions. Apart from the bottom surface, the highly aligned ions impinge on those nanowire sidewalls that possess an angle of > 90°, removing the previously built passivation layers also from there. Consequently, etching by F* can occur at positively-inclined sidewalls, reducing the sidewall angle down to 90° (see Fig. 3d).

The ICP-RIE process was also influenced by the ion density in the plasma^54,55^. A higher ICP power provides the system with a greater species flux (i.e., ion and radical) or a higher number of species per unit area for the physical bombardment. Thus, while the RF power is maintained at a constant value, the physical bombardment flux can be increased by raising the ICP power. In other words, these two power parameters (i.e., RF and ICP powers) produce a similar trend towards the nanowire profiles as depicted in Fig. 2e,f. Higher ICP power results in a decrease of sidewall angle and a rise of nanowire height (see Fig. 3e). However, it should be noticed that the nanowire height does not linearly increase. Instead, it converges to a value of ~15 µm, which can be attributed to the ARDE effect where etch rate depends on the feature size^49^. The deceleration of etch rate is related to the transport mechanism of etching species, which is more and more hindered within the developing narrow space between neighboring nanowires before the bottom surface is reached. Consequently, when a certain depth (e.g., 15 µm) is achieved, a further increase of the ICP power will not increase etch rate effectively.

In the described cryogenic ICP-RIE experiments, high-aspect-ratio Si nanowires could be achieved by tuning and optimizing the five key etching process parameters (i.e., temperature, O_2_ content, pressure, RF power, and ICP power). Here, Si nanowire arrays with a highest aspect ratio of ~20 and a smallest diameter of 830 nm were realized. Maintaining the sidewall angle at around 90° was crucial to obtain Si nanowire anodes with good verticality and length. Nanowires with sidewall angles of < 90° and > 90° were shown to collapse and randomly create black Si, respectively, during the etching process. Different Si nanowires profiles produced with various process parameters using cryogenic ICP-RIE are shown in Fig. 3a–e.

### Structural defects in dry-etched nanowires

Although the mask is intended to act as a structural template for the underlying Si, it is often found that the final lateral dimensions of etched Si are not consistent with the mask pattern. This dissimilarity comes from structural defects (e.g., undercut and bowing), which are associated with an isotropic etching behavior^56–58^. These defects become more detrimental at the small structure and cross-sectional area, such as in Si nanowire, with respect to the larger structures and trenches.

Undercut refers to the abrupt change of Si nanowire diameter directly below the mask, whereas bowing occurs as an inconsistency of lateral diameter along the Si nanowire height. The slope of bowing continuously changes with nanowire height, unlike positive (> 90° sidewall angle) or negative (< 90° sidewall angle) slopes of sidewalls that arise due to either over- or under-passivation (see Figure S2 in the Supplementary Information).

**Figure 4:**
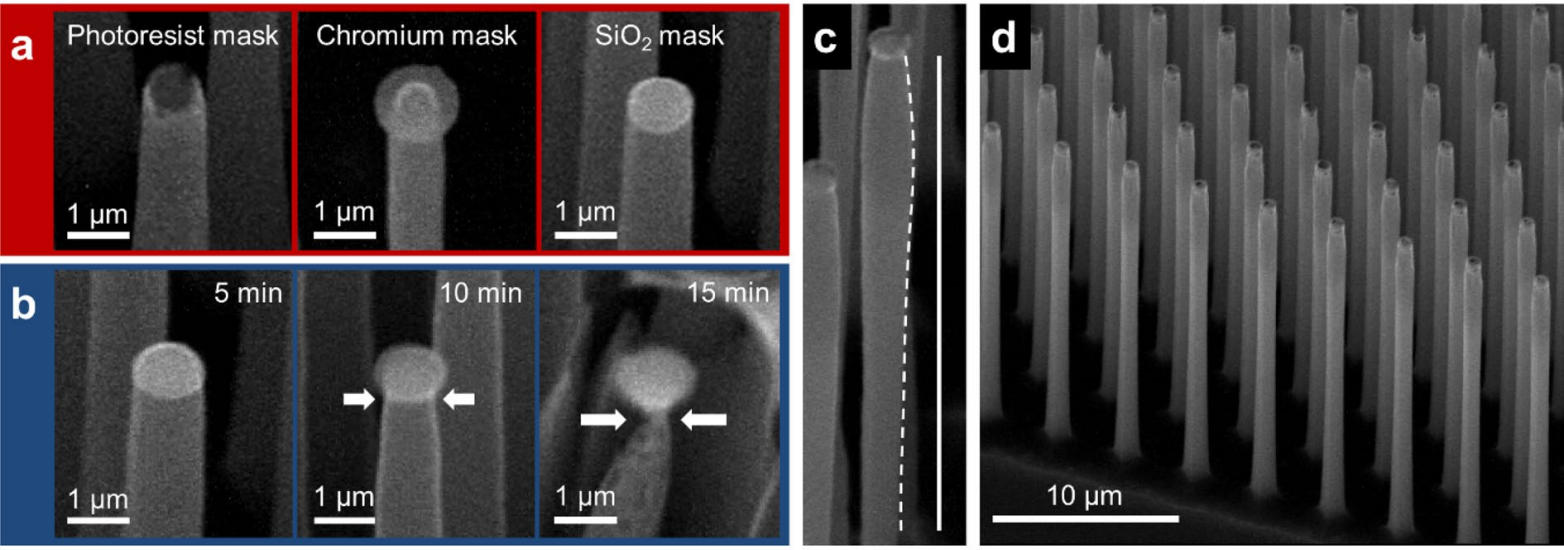
Structural imperfections in high-aspect-ratio Si nanowires affected by different mask materials during ICP-RIE at cryogenic temperature. (**a**) Undercut effects in different mask materials etched using the same etching conditions for 5 min. (**b**) Evolution of undercut in Si nanowires with a SiO_2_ mask at etching durations of 5, 10, and 15 min. (**c**) Bowing effect where the diameter of Si nanowire varies with length, forming a larger diameter at the middle compared to upper and lower parts. (**d**) Vertical Si nanowire arrays with an aspect ratio of 22.

Undercuts were observed in all Si nanowire samples etched using three different masks (i.e., photoresist, Cr, and SiO_2_) after 5 min of etch time (see Fig. 4a). For nanowire obtained with photoresist mask, the mask was removed after the etching process as a pretreatment for structural characterization. The undercut value was calculated by subtracting the initial mask diameter with the final top diameter of the nanowire below the mask, and taking it as a percentage of the initial mask diameter. Using this method, undercut values of 24.8%, 42.5%, and 16.9% were yielded for photoresist, Cr, and SiO_2_ masks, respectively. From the calculated values and the visual appearances during SEM imaging, it is evident that photoresist and SiO_2_ masks showed a relatively small undercut, while it is more severe for the Cr mask. At this point, one can suspect that the origin of the more extreme undercut of the Cr mask is due to the accumulated negative charges at the edges of the mask, which attract and bend the trajectories of positively charged ions. Moreover, due to the insulating property of photoresist and SiO_2_, the charge would be evenly distributed (i.e., not accumulated at the mask edge) producing less attractive forces to the bombarding ions. Similar behavior was also found in another study comparing Cr and chromium(III) oxide (Cr_2_O_3_) mask effects on Si nanowire undercuts during ICP etching^59^. According to that report, although Cr and Cr_2_O_3_ can create different charge accumulation behavior inducing ion attraction, the undercut phenomenon is suggested to be more influenced by the employed etching method [i.e., metal assisted plasma etching (MAPE)], in which the metal contributes to provide electrons for fluoride formation in the plasma. Hence, etching below the mask is boosted resulting in an extreme undercut as found in our case. In the MAPE process, the catalytic enhancement of etching needs a direct Si-metal interfacial contact, which is similar to liquid-phase MACE. However, not all metals that function as MACE catalysts (e.g., Au, Ag, Cr, Pt, and Cu) can be employed as MAPE catalysts^60^. Pt and Cu possess dissimilar etching behaviors when they are used in both MAPE and MACE.

To investigate the effect of etching duration to the undercut, three different samples, which had been patterned in the same photolithography batch, were etched for 5, 10, and 15 min, respectively, using the same etching parameters. The mask used for this experiment was SiO_2_, considering its high selectivity and low undercut value. From the results depicted in Fig. 4b, the undercut was more prominent after longer etching duration. Here, the major decrease of diameter only occurred at the area directly below the mask. Hence, the rest of the nanowire did not have a significant diameter reduction. This phenomenon signifies the presence of mask material, which not only acts as the protection for the underlying Si in cryogenic etching, but also has a strong effect on controlling the lateral etching behavior particularly at the area directly below the mask. If the propagation of undercut continues with time, the mask will collapse at a certain etch time, and consequently, the top area of Si nanowire will be exposed to the ion bombardment.

In Si nanowire etching, the etched area surrounds the non-etched area that has a relatively small dimension (i.e., < 1 µm). Therefore, the percentage of etched area is usually much larger than that of the non-etched area. This makes the etching more prone to failure due to two reasons. First, in the low passivation condition where etching is less anisotropic, the diameter of the nanowire reduces quickly leading to a wire collapse only after short etch time. Thus, for the same etch rate, structures that require longer etch time (e.g., high-aspect-ratio nanowires) become more challenging to be achieved. Second, during the etching process, radicals and etching products are easier to transport into and out from the sample bottom surface. This is not only because of the large etched area, but also the ability of the etching species to move at a higher degree of freedom at the bottom of the sample. Therefore, the etching dynamics becomes more complex when a deeper etching has to be performed. This complexity can result in a bowing effect as shown in Fig. 4c. Nevertheless, by fine-tuning of the etching parameters and the use of photoresist mask with simple lithography process, high-aspect-ratio Si nanowire arrays with low undercut, low bowing, and high homogeneity could be obtained (see Fig. 4d). They possess a height of 21 µm and diameters of 850–950 nm, resulting in an aspect ratio of ~22, which is considerably high for such free-standing vertical nanostructures.

### Nanowire mechanical properties probed by Raman spectroscopy

In addition to the SEM characterization of the nanowire geometry, further investigations were carried out using Raman spectroscopy to probe the mechanical properties of Si nanowires after being patterned from their bulk. This nondestructive method was previously used by another group to determine the local strain in individual indium phosphide (InP) nanowires^61^. Meanwhile, insights on the stress conditions of GaN nanowire arrays during lift-off transfer onto foreign substrates (i.e., either compressive or tensile stress) could also be gained by Raman scattering characterization^36^. In terms of Si nanowires, the former Raman study was conducted in structures obtained by electrochemical etching^62^. It was also demonstrated that the shift of Raman peak corresponds to the applied stress as well as lattice stress^63,64^.

**Figure 5:**
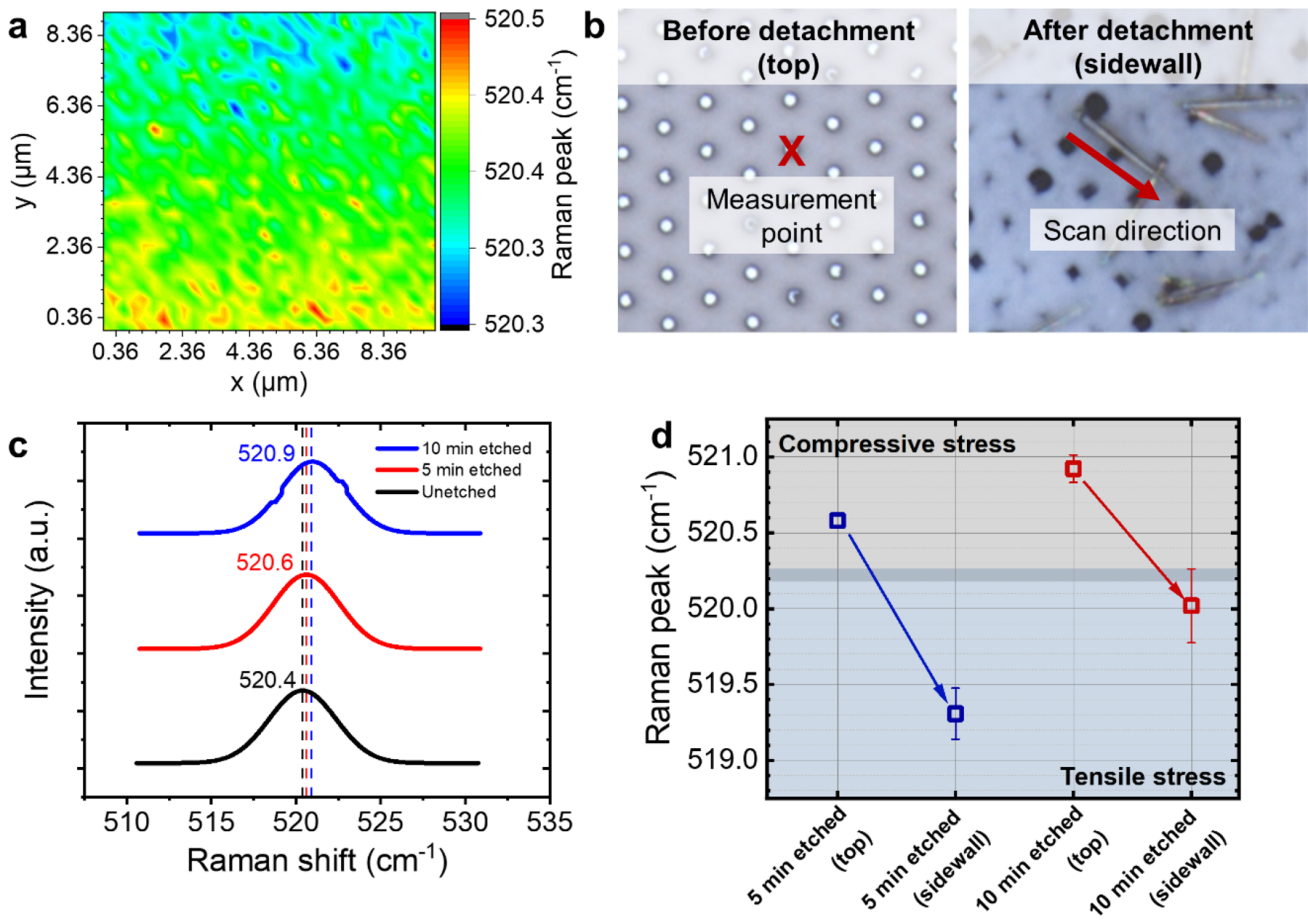
Raman measurements of Si nanowires produced by cryogenic dry etching. (**a**) Two-dimensional (2D) mapping of the dominant Raman peak at around 520.4 cm^−1^ represented by the triple degenerated optical phonon mode of unstrained planar Si, showing a homogenous distribution within ± 0.1 cm^−1^. (**b**) Two different stages of Raman characterization of the Si nanowires (i.e., before and after their detachment from the substrate). (**c**) Effect of the etch time on the Raman peak shifts. Shifts of 0.2 cm^−1^ and 0.5 cm^−1^ towards higher wavenumbers occurred after the planar Si was nanopatterned and etched for 5 and 10 min, respectively, indicating the presence of compressive lattice stress in the nanowires. Raman spectrum of planar Si (black curve) is plotted as a reference. (**d**) Difference in mechanical property of Si nanowire between top and sidewall, corresponding to compressive and tensile stresses, respectively. This indicates an anisotropy of lattice stress distribution on the surface of Si nanowire.

In our case, the Raman measurements were performed with Si nanowires and planar bulk Si as a reference. Figure 5a displays two-dimensional (2D) mapping of planar Si of an area of 9 × 9 µm^2^ with a Raman peak of ~520.4 cm^−1^ representing the triple degenerated optical phonon mode of stress-free Si as also found in other reports^65–67^. This peak value that relies within the distribution of ± 0.1 cm^−1^ is considered to be very homogenous, considering the spectral resolution of the spectroscope. To investigate the effect of etch duration on the mechanical properties of Si nanowires, two samples were fabricated with etch times of 5 and 10 min. For each sample, the measurements were carried out in two stages, i.e., before and after detachment of the nanowires from the substrate (see Fig. 5b). At the first stage, the laser beam spot was held stationary on the top of a single nanowire that were still vertically attached to the substrate. At the second stage, scanning measurements were performed along the sidewalls of individual collapsed nanowires after they were detached from the substrate. Raman peaks taken at different points along the nanowire sidewalls show homogenous wavenumbers indicating that there was no particular dependence of the Raman signal to the sidewall positions (see Figure S3 in the Supplementary Information). It should be noted that all Raman shift values presented in this work were averages over several nanowires (i.e., 2–6 wires) as listed in Table S1 in the Supplementary Information.

Compared to planar bulk Si, Si nanowire samples with longer etch time (higher structure) yield larger shifts of the Si phonon band towards higher wavenumbers as depicted in Fig. 5c. In this case, the Si phonon band of planar Si shifts from 520.4 to 520.6 cm^−1^ and 520.9 cm^−1^ when it has been nanopatterned and etched for 5 and 10 min, respectively. This systematic right shift in the Raman peak can be attributed to a decreased length of Si–Si bond corresponding to a compressive lattice stress, instead of a contribution from measurement uncertainty caused by the spectral resolution limit (see Figure S4 in the Supplementary Information)^63,64^.

When the Si nanowires were detached from the substrate (i.e., sidewalls were measured), both samples (5- and 10-min-etched Si nanowires) exhibit the same patterns, in which a shift of Si phonon band to the lower wavenumber occurred (see Fig. 5d). For the 5-min-etched Si nanowire sample, the Raman peak shows a decrease from 520.6 to 519.3 cm^−1^ when the Raman shift has been measured from top and sidewalls, respectively. A similar trend occurs in the 10-min-etched Si nanowire sample, where the Raman measurements from the top wire surface area and sidewalls result in a decrease of Raman peak from 520.9 to 520.0 cm^−1^, respectively. Furthermore, the peak wavenumbers of the sidewalls of Si nanowires are lower than that of the top positions, indicating an anisotropy of lattice stress distribution, in which top area of Si nanowires experienced compressive stress in opposite to the tensile stress presented at the sidewalls.

Besides Raman spectroscopy, which is considered as an indirect mechanical testing method, nanoindentation had been employed in our previous investigation to directly characterize the mechanical properties of Si nanowires^29,68,69^. Here, Young’s modulus and hardness of vertical Si nanowires with different crystal orientations (i.e., Si <100> , Si <110> , and Si <111>) were determined. Other studies also investigated the mechanical characteristics of horizontal Si nanowires (i.e., the nanowires that are harvested from their bulk and placed on a foreign substrate) using a similar nanoindentation approach^70,71^. The difference between the Young’s modulus of vertical and horizontal Si nanowires from those studies suggested the anisotropy of strain characteristics between axial and lateral directions, which agrees with the different strain behaviors of top part and sidewall of Si nanowires found in our Raman study.

Ultimately, such information related to different strain behaviors of Si nanowires is beneficial to analyze and optimize the mechanical stability of Si nanoanode in lithium-ion batteries during lithiation and delithiation. In the future study, Raman measurement can also be performed to differentiate the morphological changes of the Si nanowire anode during the lithiation and delithiation, as well as interphase formation from electrolyte components^72^.

### Lithium-ion battery integrating silicon nanowire anode

**Figure 6:**
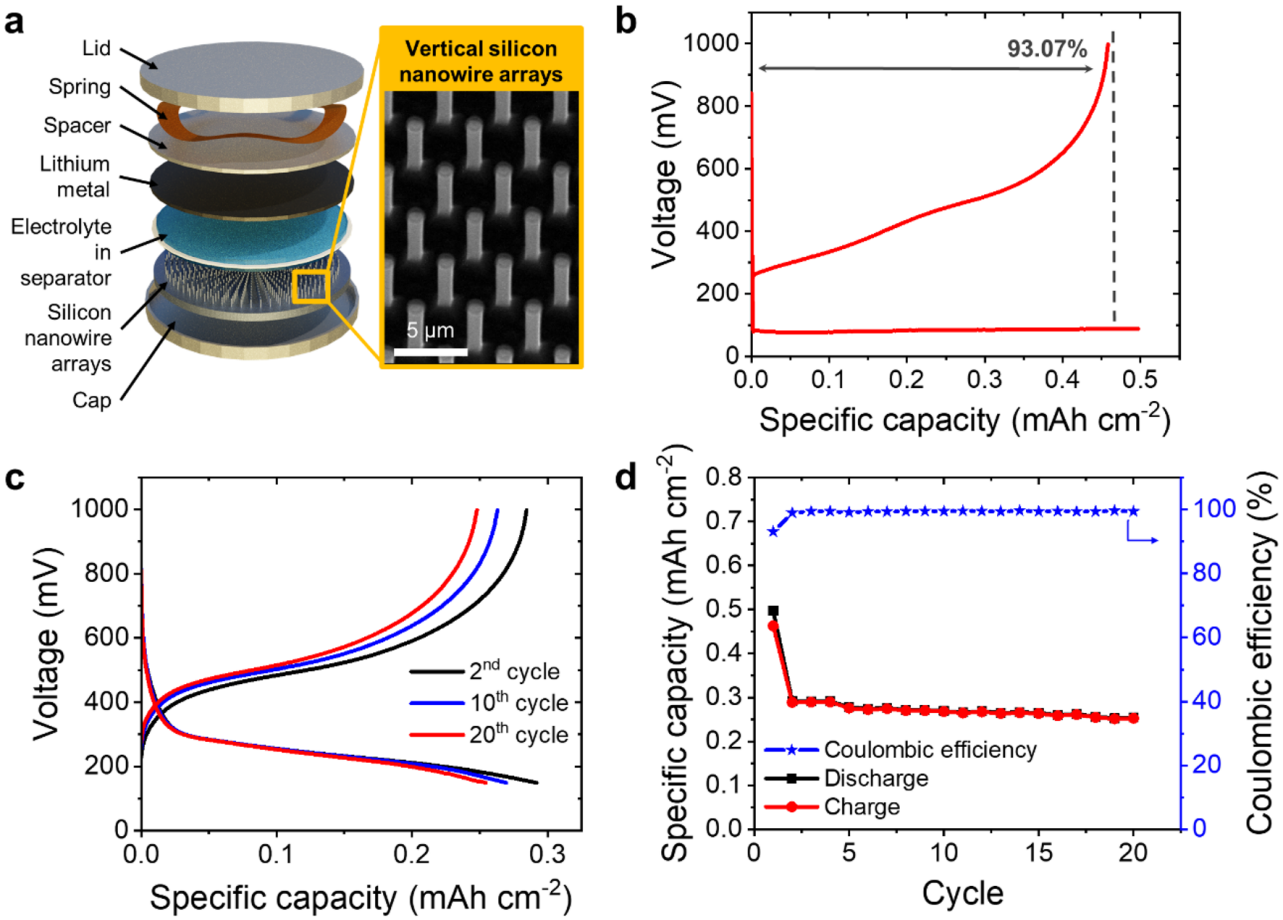
Li-ion battery with Si nanowire anode and its electrochemical properties. (**a**) Stacking order of the half-cell battery used in the testing where vertically aligned Si nanowire arrays served as one of the electrodes. (**b**) Galvanostatic discharge–charge profile of Si nanowire array at the prelithiation cycle, showing initial Coulombic efficiency (ICE) of 93.07%. (**c**) Galvanostatic discharge–charge curves of Li-ion batteries with Si nanowire array electrode at three different cycles (i.e., 2nd, 10th, and 20th cycles). (**d**) Discharge–charge capacity and the respective Coulombic efficiency of the Li-ion battery with Si nanowire array electrode for the first 20 cycles.

The electrochemical performance of the Si nanowire array for lithium-ion battery application was investigated in a half-cell battery configuration. The Si nanowire arrays, Li metal, and other battery components were first assembled into a coin cell (see Fig. 6a). In this configuration, the Si nanowire array and Li metal serve as a positive electrode (cathode) and a counter electrode (anode), respectively. The battery was then exposed to repeated discharge and charge cycles by applying a constant current density, in which the changes of capacity and voltage were recorded simultaneously. When the battery is discharged, an external load drives the electrons from Li metal through an external circuit to the Si nanowire. At the same time, lithium ions (Li^+^) are transported from the Li metal to the Si nanowire through the electrolyte. The charging process occurs when the external potential is applied to the battery. Li^+^ are shuttled back to the Li metal through the electrolyte, while the electrons are transported via the external circuit.

During the prelithiation cycle, Si nanowire arrays with a diameter of ~1 μm and a height of ~3.9 μm reached specific discharge and charge capacities of 0.497 mAh cm^−2^ and 0.463 mAh cm^−2^, respectively, giving an initial Coulombic efficiency (ICE) of 93.07% (Fig. 6b). The prelithiation step is required to load Li^+^ from the Li metal onto Si nanowire arrays^73^. During the prelithiation, solid electrolyte interphase (SEI) is formed consuming the active Li. Consequently, the Si nanowire experiencing a capacity drop right after the prelithiation cycle. After the prelithiation step, the charging capacity drops from 0.285 to 0.248 mAh cm^−2^ at the end of the 20th cycle, retaining 87.17% of its charging capacity (Fig. 6c). When discharged, the first cycle after the prelithiation step shows a discharge capacity of 0.30 mAh cm^−2^, which reduces to 0.25 mAh cm^−2^ at the end of the 20th cycle, corresponding to 16.7% capacity fading. Here, the normalization of the capacity to area was used instead of the capacity to mass because the integrated anode was the whole bulk wafer with the vertically aligned nanowire on top of it (i.e., the nanowires were not harvested or removed from the bulk silicon). Since the mass of the bulk, which does not contribute to the capacity, is much larger compared to the surfaces, mass-related specific capacity is not an appropriate metric and surface-related values are given instead.

In this study, we tested only the first several cycles (i.e., 20 cycles) to investigate the behavior of the Si nanowire anode, in which the typical drop of capacity at the prelithiation step was observed. Indeed, to gain a more representative insight on cycling performance, more cycling test numbers (e.g., 250, 500, 1000, or more cycles) are necessary that are commonly conducted for battery performance qualification and verification^74–76^. As this article has focused more on the fabrication technique of vertical Si nanowires using ICP-RIE, long-term stability of the anode has not been carried out and therefore will be addressed in our next studies. Figure 6d depicts the galvanostatic charge–discharge curves that show the charge–discharge capacities and the corresponding Coulombic efficiency of each cycle. The Coulombic efficiency of the Li-ion battery with Si nanowire electrode is stable at > 99% until the end of the 20th cycle.

It should be noted that the electrochemical test presented here was done using Si nanowires with low aspect ratio (i.e., 3.9:1). The test, which was carried out for 20 cycles already showed a degradation of discharge capacity of 16.7% (i.e., from 0.30 to 0.25 mAh cm^−2^). This phenomenon could be attributed to the degradation of Si nanowires with similar geometry as found in some studies^10,77^. This result indicates that the geometry of the nanowires used for this test needs to be optimized (e.g., by reducing the diameter) to better facilitate the volume change upon longer cycles.

Moreover, the test was done only in a low current rate (i.e., 0.06 mA cm^−2^). At higher current rates (e.g., in a rate capability test), a lower capacity of lithium-ion batteries using Si nanowires anode was reported^8,78,79^. In this context, the potential of heavily-*n*-doped Si nanowires for ultrafast charging has been demonstrated, showing the importance of conductivity of silicon anodes in battery performance^16,80^. Typically, cycling tests at different current rates have to be conducted to observe this characteristic. As in our current work, we still utilized moderately *n*-doped Si nanowires and the battery was not tested at higher current rates yet, ultrafast charging could not be confirmed so far. In the next step, therefore, improvements will be executed by employing high-aspect-ratio Si nanowires with higher doping concentration (of ~10^19^ cm^−3^ with respect to ~10^15^ cm^−3^ in the present study) and simultaneously conducting cycling measurements at higher current rates.

Despite the successful proof-of-concept demonstration of the half-cell battery with Si nanowire anode, solid strategies should be dedicated to enhance its performance (e.g., the use of higher, smaller diameter, and denser nanowire arrays). The fabrication technique presented in this study could be used to produce high-aspect-ratio Si nanowires with 21 µm length using an established process. The larger surface area at higher aspect ratio is expected to facilitate more area for lithium insertion and thus to enable a larger storage capability. Improvement of the electrochemical performance was planned by reducing the wire diameter down to < 200 nm to obtain a larger aspect ratio and at the same time suppressing the swelling effect of Si upon cycling. More material characterizations are also still required to understand the phenomena occurring during battery operation, especially to compare the conditions of the Si nanowire anode before and after cycling tests at different current rates.

## Conclusions

Vertical Si nanowire arrays have been successfully fabricated by inductively coupled plasma reactive ion etching (ICP-RIE) at cryogenic temperature and subsequently utilized as an anode for half-cell lithium-ion battery. Besides considering three different mask materials (i.e., photoresist, chromium (Cr), and silicon dioxide (SiO_2_)), several key etching parameters were tuned and optimized resulting in homogenous Si nanowires with a high aspect ratio of up to 22 and smooth perpendicular sidewalls. Among the investigated mask materials, the SiO_2_ mask has exhibited good selectivity and the lowest effect of undercuts on the nanowires demonstrating its suitability for realizing ICP-RIE-based Si anodes. By means of Raman spectroscopy, compressive stress was detected in the Si nanowires, which was higher when they possessed greater height resulting from longer etching duration during nanopatterning. Measurement from the top and sidewall of the nanowires revealed an anisotropy of lattice stress of those facets, indicating compressive and tensile stresses, respectively. From the electrochemical characterization, a developed battery containing a Si nanowire anode has exhibited stable performance for 20 cycles at a capacity of around 0.25 mAh cm^−2^. Further improvement of Si nanowire battery performance could be expected by implementing several strategies (e.g., reducing the nanowire diameter, using nanowires with higher doping concentration, fabricating a hierarchical structure, and applying a surface functionalization on the nanowire). This study shows that cryogenic dry etching could be a versatile fabrication route for Si nanowire anodes, especially when verticality and homogeneity of the structures have to be maintained.

## Methods

### Nanowire patterning

The basic sample materials used in all experiments were *n*-type Si(100) wafers with moderate phosphorous doping (electrical resistivity, *ρ* = 5–10 Ωcm) from Siegert Wafer GmbH, Aachen, Germany. Si nanowire arrays were fabricated by firstly applying circular patterns to Si wafers as a template using photolithography. Depending on the used mask types (i.e., photoresist, Cr, and SiO_2_), different treatments were applied to deposit the mask layers (see Fig. 1). For the photoresist mask, the patterning material was straightly deposited in the photolithography process. Prior to patterning, Si samples were cleaned using acetone in an ultrasonic bath and subsequently dried using a nitrogen blow. Surface pretreatment to enhance the adhesion of photoresist was performed by applying hexamethyldisilazane (HMDS) to the sample on a hotplate at 115 °C. Within 10 min after HMDS treatment, AZ 5214 E photoresist diluted in AZ EBR (1:1) was spin-coated at 3000 rpm for 35 s, followed by pre-baking at 110 °C for 50 s. The pattern from a positive mask template was then transferred using MJB4 mask aligner from SÜSS MicroTec SE, Garching, Germany by exposing the sample to UV light generated by a 210 W Hg lamp for 13 s. Finally, the undesired photoresist was removed by dipping the sample into AZ 726 MIF developer for 25 s.

For the Cr hard mask, fabrication was carried out similar to that of the photoresist mask. However, after exposure, a heating treatment was applied at 125 °C for 90 s and subsequently followed by flood-exposure of the entire sample surface by UV light for 64 s. This process was performed to inverse the image since AZ 5214 E is an image reversal photoresist. Development by AZ 726 MIF was done for 20 s to dissolve the unwanted photoresist. Then, a 100 nm thick Cr film was deposited using an electron beam evaporator. To complete the pattern preparation, a lift-off process was executed by dipping the sample into acetone in an ultrasonic bath for 30 s. Hence, the Cr layer that was deposited on the photoresist mask could be removed leaving a circular Cr thin film pattern on the Si surface.

Fabrication of the SiO_2_ hard mask began with thermal oxidation at 1100 °C inside a furnace for 35 min to grow a 130 nm thick SiO_2_ film on top of the Si wafer. Afterwards, a 350 nm thick photoresist mask was deposited to form the circular pattern using the same procedure as for the fabrication of the photoresist mask. The pattern transfer was then performed by immersing the sample into hydrofluoric acid (HF) for 2 min to remove the undesired SiO_2_ layer and subsequently dipping it into acetone to remove the photoresist mask. In this process, the photoresist mask acted as a cover for the underneath SiO_2_. The remaining SiO_2_ has a circular pattern with a slightly smaller diameter compared to the photoresist mask due to underetching.

### Cryogenic dry etching

The inductively coupled plasma reactive ion etching (ICP-RIE) of Si nanowires was conducted using a cryogenic SI 500C plasma etcher from SENTECH Instruments GmbH, Berlin, Germany. Unless stated otherwise, the recipe used by default involves several constant parameters (i.e., temperature = −95 °C, pressure = 1.0 Pa, O_2_ flow = 13 sccm, SF_6_ flow = 118 sccm, ICP power = 500 W, RF power = 6 W (RF bias = −12 V), and etch time = 5 min). Thermally conductive oil was used between the Si samples and the sample holder to ensure efficient heat transfer during the cryogenic cooling. After etching, the oil was removed from the samples using acetone, which simultaneously also strips a photoresist mask off the Si surface.

The time required for etching using cryogenic ICP-RIE is inversely proportional to the etching rate and proportional to the expected length of nanowires. We have demonstrated the production of ~15 µm nanowires in 5 min etching time which corresponds to an etching rate of ~3 µm/min. Moreover, since an extra time is required for cooling from room to cryogenic temperature, subsequent process of samples is beneficial to have a more efficient production. Meanwhile, the number of nanowire produced at each batch depends on the patterning process. Here, we use photolithography with a photomask by the size of 2″ wafer with a nanowire areal density of 4.8 × 10^6^ cm^−2^.

### Structural and material characterizations

Scanning electron microscopy (SEM) images of Si nanowires were captured using a Leica Cambridge S360FE SEM at room temperature. The samples were 45°-tilted to obtain three dimensional (3D) images of the nanowires. The height of nanowires was calculated according to trigonometry identity (i.e., ${h}_{actual}={h}_{SEM}\sqrt{2}$). Raman spectroscopy was carried out using a Renishaw inVia™ Qontor Raman microscope with a laser wavelength of 532 nm and a 100× objective lens with numerical aperture of 0.85. The grating used in the measurements has a spectral resolution of 0.8 cm^−1^ while the effective pinhole has a diameter of ~20 µm.

### Battery fabrication and test

For our experiments, a half cell of CR2016 coin cell battery was assembled using 1 × 1 cm^2^ silicon wafer with the thickness of 525 µm as the anode, in which it had been firstly patterned with nanowires on its top surface. In other words, the height of nanowires and the thickness of the remaining bulk are 3.9 µm and 521.1 µm, respectively. It should be noted that no detachment of Si nanowires from the substrate was performed. Thus, the anode was not further mixed with binders, carbon, or other additives, which is typically done for common silicon nanowire anode in other studies^13,81^. Therefore, mass loading of this anode is equivalent to the mass of silicon sample itself (124.11 mg cm^-2^). Consequently, the capacity per surface area of each sample was calculated with respect to the area of the sample (i.e., 1 × 1 cm^2^).

Electrochemical performance of the Si nanowire arrays with an area of 1 × 1 cm^2^ was performed by assembling it into a half cell of CR2016 coin cell battery. The employed electrolyte consisted of 1 M solution of LiPF_6_ diluted in a mixture of ethylene (EC), dimethyl carbonate (DMC), and diethyl carbonate (DEC) with a 1:1:1 volume ratio. A polypropylene separator (Cellgard 2400) was used to isolate the Si electrode from a lithium metal counter electrode. Those components were stacked together inside a glove box (Vygor) to maintain H_2_O below 1.0 ppm and O_2_ at 0 ppm. They were stored for 10 h to ensure a uniform wetting of the electrolyte. Prelithiation was done by discharging the cell from 1.0 to 0.01 V at a constant current density of 0.05 mA cm^−2^ for 10 h to ensure the formation of the surface electro-active region. Capacity measurement was carried out using galvanostatic charge–discharge in Neware battery tester for 20 cycles at the current density of 0.06 mA cm^−2^ between 0.15 and 1.0 V at room temperature.

## Supplementary Information

Supplementary Information.

## Data Availability

All data supporting the findings of this study are available within the article and Supplementary Information file.
