# Supplementary material for: Versatilely tuned vertical silicon nanowire arrays by cryogenic reactive ion etching as a lithium-ion battery anode
Source: Sci Rep. 2021 Oct 5;11:19779. doi: 10.1038/s41598-021-99173-4 (PMC8492725; doi:10.1038/s41598-021-99173-4)
Supplement: Supplementary file 1 — Supplementary Information. [file 41598_2021_99173_MOESM1_ESM.docx]

Supplementary Information

Versatilely tuned vertical silicon nanowire arrays by cryogenic reactive ion etching as a lithium-ion battery anode

**Andam Deatama Refino^1,2,3,*^, Nursidik Yulianto^1,2,4^, Iqbal Syamsu^1,2,5^, Andika Pandu Nugroho^6^, Naufal Hanif Hawari^6^, Alina Syring^1,2^, Evvy Kartini^7^, Ferry Iskandar^8^, Tobias Voss^1,2^, Afriyanti Sumboja^6^, Erwin Peiner^1,2^, Hutomo Suryo Wasisto^1,2,9,*^**

^1^ Institute of Semiconductor Technology (IHT), Technische Universität Braunschweig, Hans-Sommer-Straße 66, Braunschweig 38106, Germany

^2^ Laboratory for Emerging Nanometrology (LENA), Technische Universität Braunschweig, Langer Kamp 6, Braunschweig 38106, Germany

^3^ Engineering Physics Program, Institut Teknologi Sumatera (ITERA), Jl. Terusan Ryacudu, Way Huwi, Lampung Selatan, Lampung 35365, Indonesia

^4^ Research Center for Physics, Indonesian Institute of Sciences (LIPI), Jl. Kawasan Puspiptek No. 441-442, South Tangerang 15314, Indonesia

^5^ Research Center for Electronics and Telecommunication, Indonesian Institute of Sciences (LIPI), Jl. Sangkuriang - Komplek LIPI Gedung 20, Bandung 40135, Indonesia

^6^ Material Science and Engineering Research Group, Faculty of Mechanical and Aerospace Engineering, Institut Teknologi Bandung, Jl. Ganesha 10, Bandung 40132, Indonesia

^7^ Center for Science and Technology of Advanced Materials, National Nuclear Energy Agency (BATAN), South Tangerang 15314, Indonesia

^8^ Department of Physics, Faculty of Mathematics and Natural Science, Institut Teknologi Bandung, Jl. Ganesha 10, Bandung 40132, Indonesia

^9^ PT Nanosense Instrument Indonesia, Umbulharjo, Yogyakarta 55167, Indonesia

* Corresponding authors.

E-mails: [andam.refino@tu-braunschweig.de](mailto:andam.refino@tu-braunschweig.de) (A.D.R.); [h.wasisto@nanosense-id.com](mailto:h.wasisto@nanosense-id.com) (H.S.W.)

Contents

[1. Measurement of Si nanowire geometry S-2](#_Toc76319303)

[2. Structural defects in Si nanowire S-3](#_Toc76319304)

3. [Raman characterization of Si nanowire sidewall S-4](#_Toc76319305)

[4. Detailed Raman peak characteristics of Si nanowires S-5](#_Toc76319307)

# Measurement of Si nanowire geometry


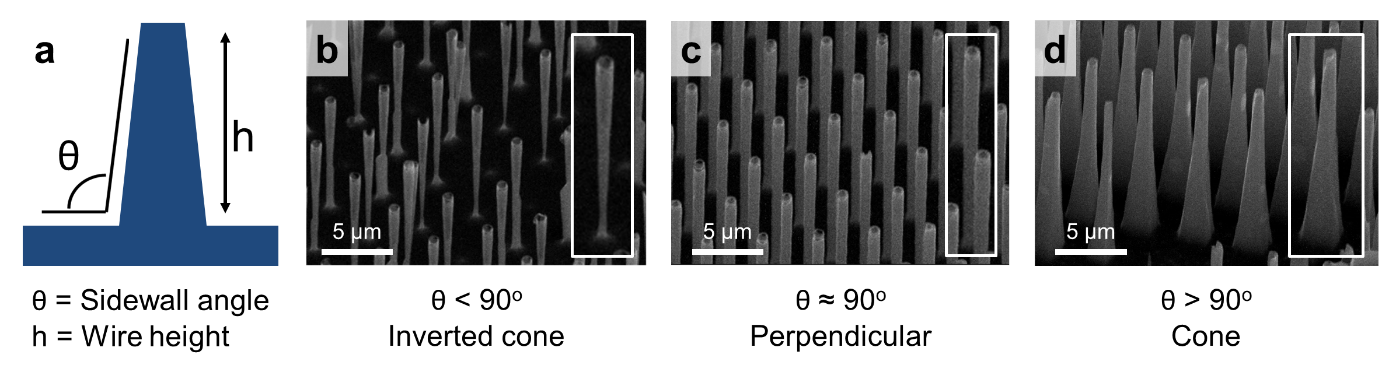


**Figure S1** **Measurement of Si nanowire height and sidewall angle**. **a** Schematic diagram indicating measured geometry parameters of Si nanowires (i.e., sidewall angle (θ) and wire height (h)). Scanning electron microscopy (SEM) images of Si nanowires with different sidewall angle classifications: **b** inverted cone (negative profile), **c** perpendicular, and **d** cone shapes (positive profile).

# Structural defects in Si nanowire


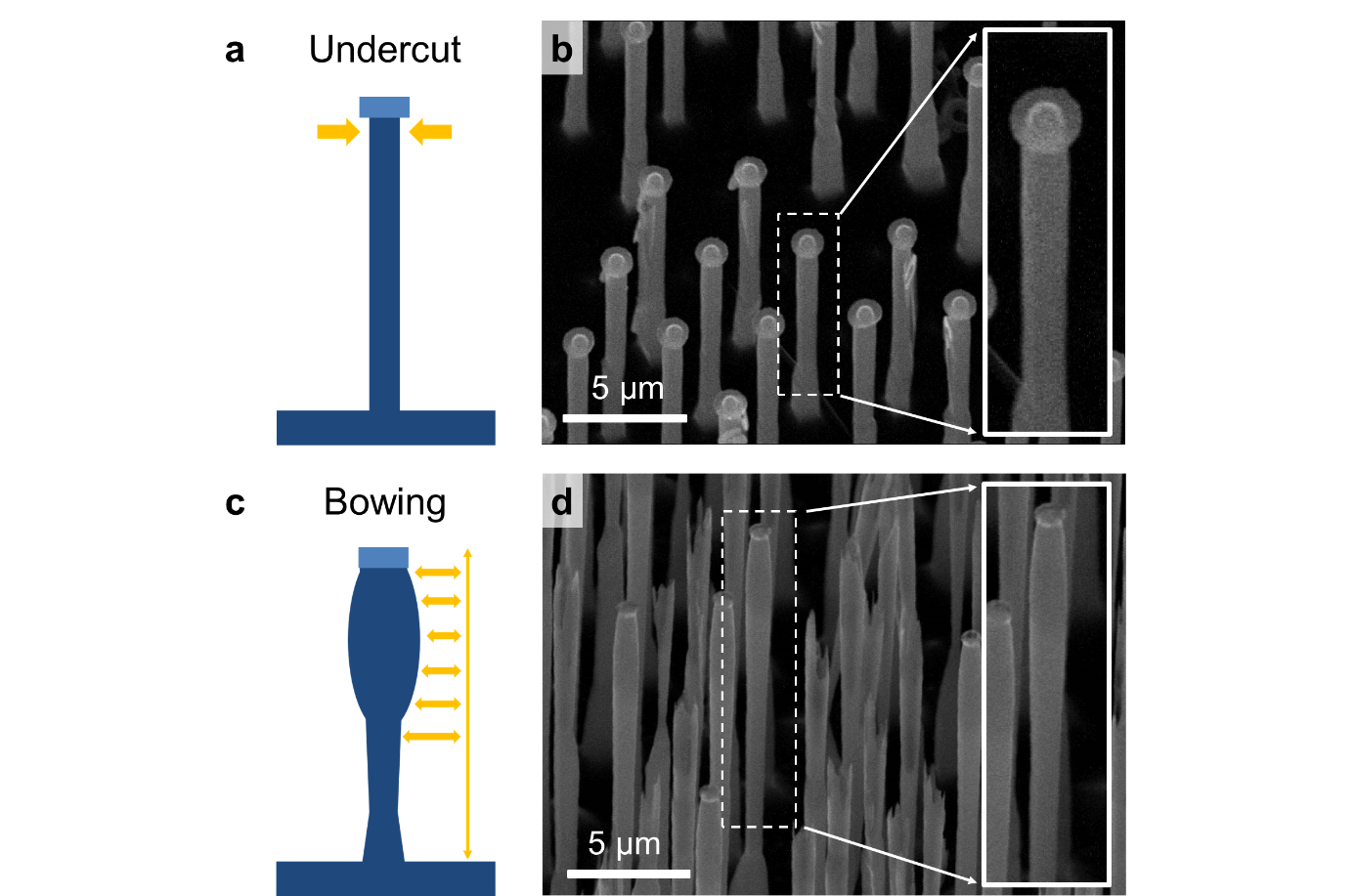


**Figure S2** **Structural defects in Si nanowire**. Schematic diagrams describing: **a** undercut and **c** bowing. Scanning electron microscopy (SEM) images of Si nanowires with structural defects from cryogenic ICP-RIE: **b** undercut and **d** bowing.

# Raman characterization of Si nanowire sidewall


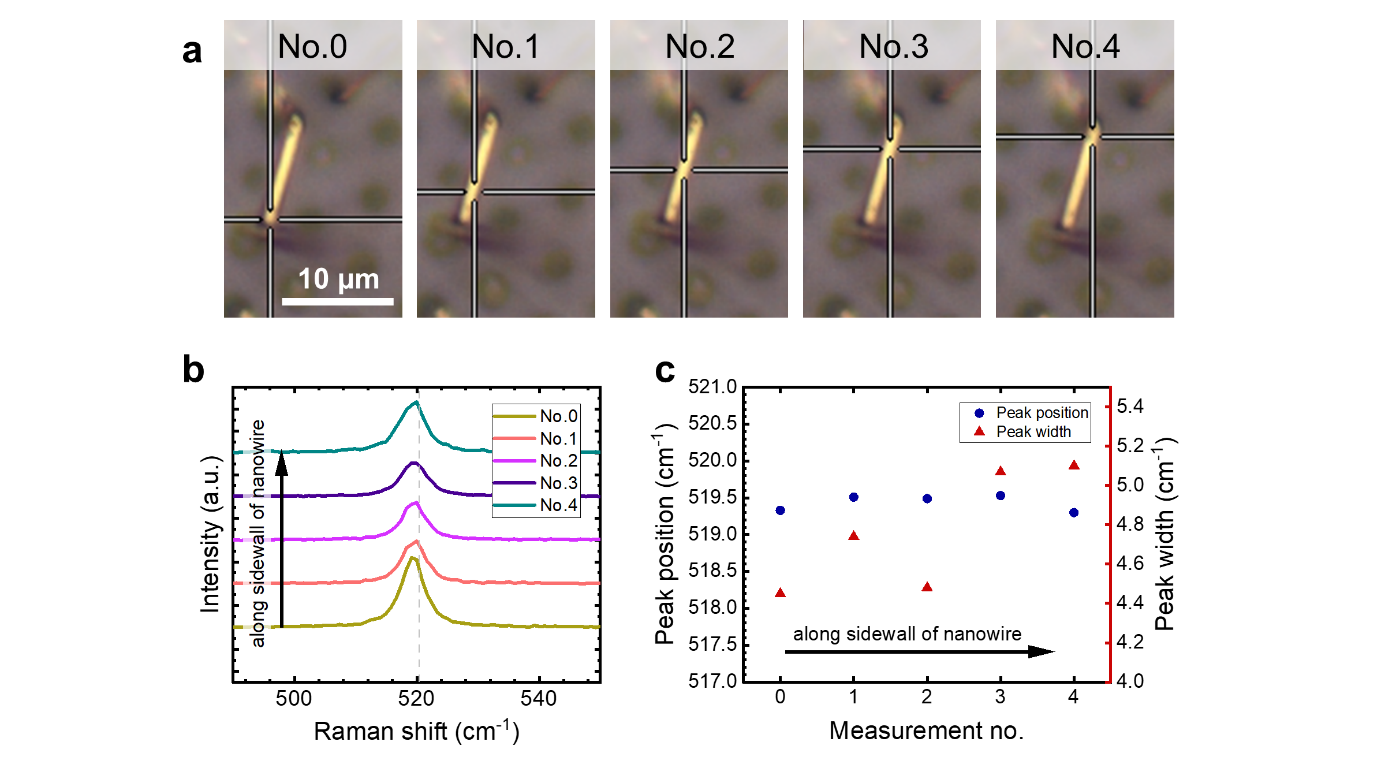


**Figure S3** **Raman characterization of a single detached Si nanowire along its sidewall.** **a** Optical microscope images of the detached Si nanowire sidewall showing five probed measurement points (indicated by the crosshairs, diameter of laser beam spot = ~1 µm). **b** Raman shift of each measurement point (i.e., No. 0 – 4). **c** Raman peak position and full width at half maximum (FWHM) of each measurement point demonstrating homogenous wavenumbers at all measurement points.

# Detailed Raman peak characteristics of Si nanowires

**Table S1** **Summary of Raman peak positions for both as-etched and detached Si nanowires at all considered measurement points.**

| **Measure-ment point/ number** | **Raman peak position (cm^-1^)** | | | | | | |
| --- | --- | --- | --- | --- | --- | --- | --- |
|  | **As-etched**  **(Top)** | | **Detached**  **(Sidewall)** | | | | |
|  | **5 min etched** | **10 min etched** | **5 min etched (1)** | **5 min etched (2)** | **5 min etched (3)** | **10 min etched (1)** | **10 min etched (2)** |
| 1 | 520.61 | 521.00 | 519.33 | 519.10 | 519.52 | 519.93 | 520.07 |
| 2 | 520.54 | 520.80 | 519.51 | 519.11 | 519.38 | 519.92 | 520.20 |
| 3 | 520.64 | 521.00 | 519.49 | 519.11 | 519.29 | 519.99 | 519.48 |
| 4 | 520.54 | 520.94 | 519.53 | 519.15 |  | 520.07 | 519.99 |
| 5 |  | 521.00 | 519.30 | 519.06 |  | 519.24 | 520.20 |
| 6 |  | 520.80 |  | 519.42 |  | 519.98 | 520.14 |
| 7 |  |  |  |  |  | 520.22 | 520.17 |
| 8 |  |  |  |  |  | 520.03 | 520.17 |
| 9 |  |  |  |  |  | 520.02 | 520.25 |
| 10 |  |  |  |  |  | 520.05 | 520.27 |
| 11 |  |  |  |  |  | 519.78 | 520.26 |
| **Average** | **520.58**  **± 0.05** | **520.92**  **± 0.10** | **519.31**  **± 0.17** | | | **520.02**  **± 0.25** | |


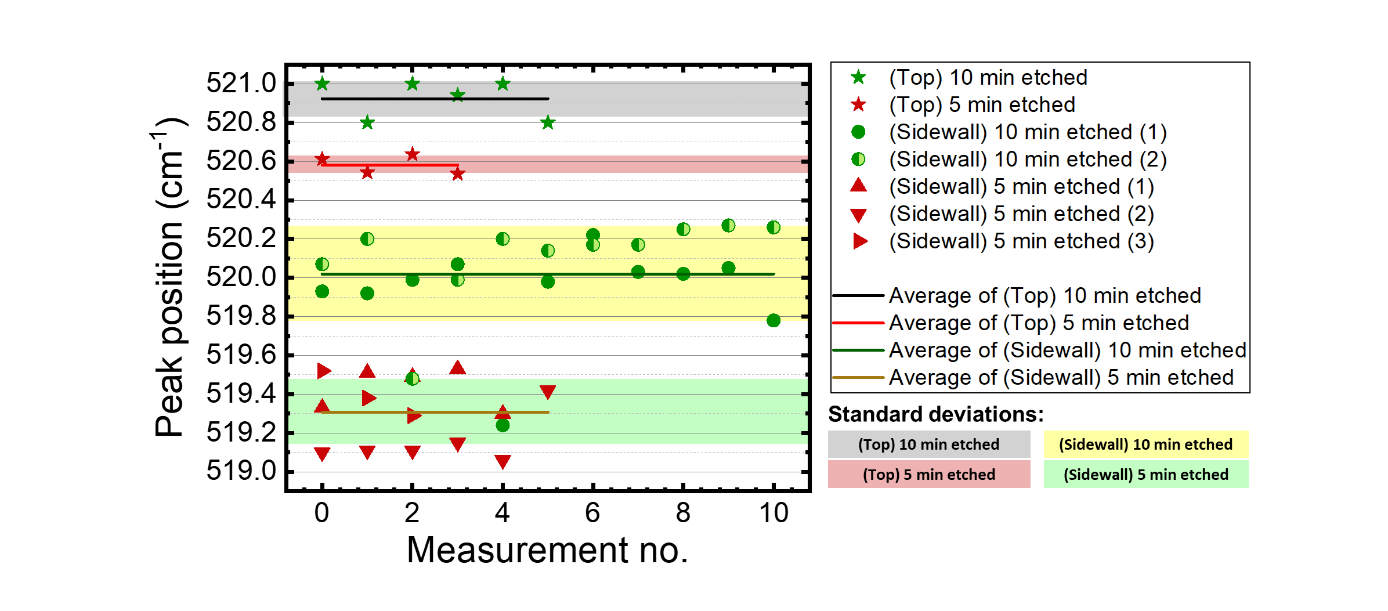


**Figure S4** **Scatter plot of Raman peak positions.** The average values of all Raman measurements taken from the top and sidewalls of nanowires etched for different times (5 and 10 minutes) as well as their respective standard deviations, indicating highly precise metrology.

Data in the main manuscript were obtained by averaging Raman peaks at different positions on the samples as shown in **Table S1** and illustrated in **Figure S4**. For the as-etched nanowires, data were taken with 4 and 6 different nanowires from the samples that were etched for 5 and 10 minutes, respectively. For the detached nanowires, data were taken with 3 and 2 different nanowires from the samples that were etched for 5 and 10 minutes, respectively. It should be noted that each detached nanowire had different measurement points along its sidewall.
